# Development of DNA and mRNA-LNP vaccines against an H5N1 clade 2.3.4.4b influenza virus

**DOI:** 10.1128/jvi.00795-25

**Published:** 2025-07-16

**Authors:** Rebecca A. Leonard, M. Ariel Spurrier, Samantha Skavicus, Zhaochen Luo, Brook E. Heaton, Rachel L. Spreng, Jiaqi Hong, Fan Yuan, Nicholas S. Heaton

**Affiliations:** 1 Department of Molecular Genetics and Microbiology, Duke University School of Medicine 12277 , Durham, North Carolina, USA; 2 Duke Human Vaccine Institute, Duke University School of Medicine 12277 , Durham, North Carolina, USA; 3 Department of Biomedical Engineering, Duke University 242238, Durham, North Carolina, USA; 4 Duke Center for Virology, Duke University School of Medicine 12277 , Durham, North Carolina, USA; St. Jude Children's Research Hospital, Memphis, Tennessee, USA

**Keywords:** H5N1 influenza, mRNA-LNP vaccines, DNA vaccines, influenza virus

## Abstract

Effective vaccines are an important public health tool which may be needed to combat the emerging, highly pathogenic H5N1 avian influenza viruses currently circulating in cattle and poultry in the United States. While nucleic acid-based vaccines such as mRNA-lipid nanoparticles (LNPs) have several potential advantages during a viral epidemic compared to traditional seasonal influenza vaccines, their utility and efficacy against H5N1 viruses remain incompletely defined. Here, we developed novel DNA- and mRNA-LNP-based vaccines encoding both hemagglutinin (HA) and neuraminidase (NA) proteins from the human-isolated highly pathogenic avian influenza H5N1 strain, A/Texas/37/2024, in a single open reading frame. This dual-antigen expression approach elicited strong protective immune responses targeting both the HA and NA proteins and provided complete protection against lethal viral challenges in a murine model. The pre-clinical data described in this work suggest that these multi-valent, adaptable, and scalable vaccine approaches may represent practical and rapid solutions to mediate robust protection from emerging zoonotic influenza virus threats.

**IMPORTANCE:**

Vaccines capable of protecting from infection with the H5N1 influenza viruses actively circulating in dairy cattle could be deployed to protect livestock and potentially also be used to protect human health. Here, we describe the development of protective DNA and mRNA-lipid nanoparticle vaccines targeting hemagglutinin and neuraminidase proteins from the highly pathogenic avian influenza (HPAI) H5N1 A/Texas/37/2024 virus and show that they are both protective against severe morbidity and mortality in a mouse model. Thus, the vaccines described in this work represent effective approaches to limit the current circulation of H5N1 viruses in animals and may represent practical solutions for human vaccination in the event of sustained human transmission of HPAI H5N1 viruses.

## INTRODUCTION

Since early 2024, highly pathogenic H5N1 avian influenza viruses of the 2.3.4.4b clade have been widely circulating in dairy cattle, poultry, and wild birds (1). While similar viruses have previously been detected in North American birds, their sustained transmission in livestock represents a concerning evolutionary shift (2, 3). B3.13 genotype 2.3.4.4b viruses, exemplified by the A/Texas/37/2024 strain, have infected over 950 cattle herds across 17 states, leading to viral shedding in milk and reduced milk production (1, 4, 5). Meanwhile, D1.1 genotype H5N1 viruses have been spreading in poultry flocks, threatening egg supplies, and displaying increased human exposure among farm workers (1, 4, 5). Alarmingly, 70 human cases of 2.3.4.4b H5N1 infection have been confirmed, primarily in individuals with prior exposure to infected animals, but notably, three cases had no known exposure (6). While most infections have resulted in mild respiratory symptoms or conjunctivitis, one patient in Louisiana, infected after contact with a backyard flock, developed severe disease and later died (7–9). Although human-to-human transmission has not been observed, the virus’s continued spread in animals heightens the risk of mutations that could facilitate sustained transmission among humans. Thus, there is a critical need for effective interventions to limit H5N1 viral transmission and mitigate the risk of a future influenza pandemic.

One of the most effective tools for controlling emerging viral threats is vaccination, yet traditional vaccine platforms face limitations in their ability to rapidly respond to novel pathogens. Platforms used for seasonal influenza vaccines, such as inactivated and live-attenuated vaccines, require extensive development time, complex production pipelines, and large-scale manufacturing, all of which delay deployment during outbreaks (10, 11). In contrast, nucleic acid-based vaccines, including DNA and mRNA platforms, offer an adaptable and scalable solution for pandemic preparedness due to their adaptability to varied genetic sequences. Sequence-agnostic mRNA vaccines, formulated with lipids to generate lipid nanoparticles (LNPs), generally enable faster production, stronger immune responses, and more efficient delivery, making them ideal candidates for human delivery during real-world outbreak response, as was exemplified during the COVID-19 pandemic (12–14).

Seasonal influenza vaccines primarily focus on inducing hemagglutinin (HA)-specific immune responses, as HA-targeted antibodies are crucial for viral neutralization and protection (15–17). A singular focus on HA, however, overlooks the impactful contributions of neuraminidase (NA)-directed immune responses, which have been shown to lower viral loads, reduce transmission, and lead to milder disease outcomes (18–20). To elict immune responses to both HA and NA, we previously developed an improved seasonal mRNA-LNP vaccine platform capable of expressing multiple viral glycoproteins from a single mRNA molecule. Application of this technology to seasonal influenza subtypes (influenza A H1N1 and H3N2 subtypes, influenza B (IBV) Yamagata and Victoria lineages) in a quadrivalent vaccine format elicited robust immune responses; however, the approach has not been tested with emerging or zoonotic influenza viruses. Given the continued spread and evolution of H5N1 in mammals, a dual-glycoprotein vaccine strategy could be a crucial step in developing more effective countermeasures against emerging influenza pandemic threats.

Here, we develop novel nucleic acid-based vaccines against the zoonotic H5N1 strain, A/Texas/37/2024, which was isolated from an infected dairy worker in Texas and thus represents an H5N1 strain with a demonstrated ability to cause disease in humans (21). Both the DNA- and mRNA-LNP-based vaccines make use of a single open reading frame (ORF) genetic configuration to deliver the genetic material of the influenza HA and NA proteins from a single molecule of either DNA or RNA. After validating that the configuration allowed for proper delivery and expression of both proteins in cells, we showed that the DNA-based vaccines are immunogenic and protective in mice, conferring full protection from viral challenge and disease. We then translated this design into an mRNA-LNP vaccine, which was shown to elicit functionally neutralizing antibodies and provide complete protection against lethal H5N1 challenge in mice. These findings show that both DNA and RNA-based nucleic acid vaccine platforms represent promising approaches for rapidly countering emerging zoonotic influenza viruses both in their animal hosts and in preparation for possible human adaptation.

## RESULTS

**Fig 1:**
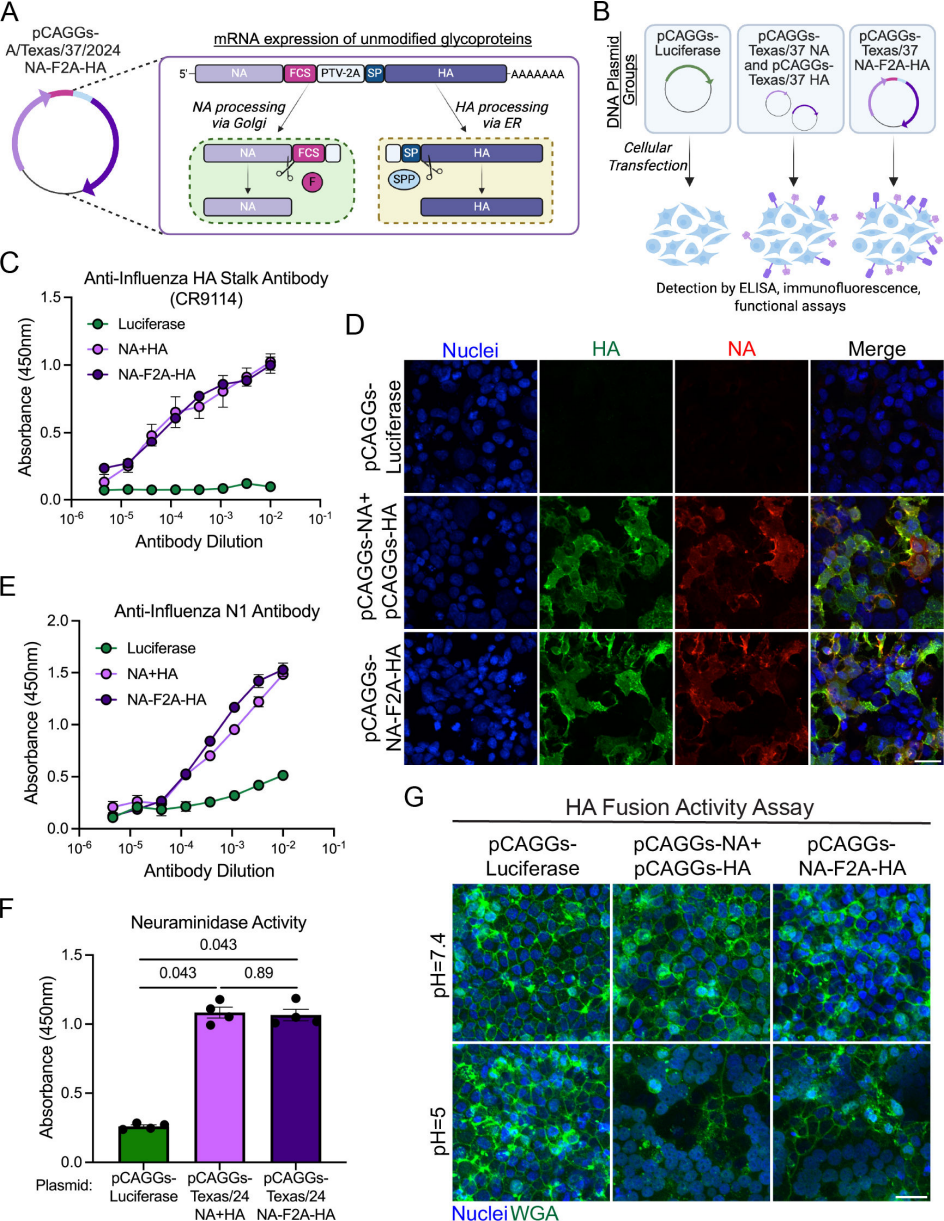
Expression of the H5 and N1 proteins from A/Texas/37/2024 from a DNA vector. (**A**) Schematic of pCAGGs-NA-F2A-HA genetic design and expression of unmodified glycoproteins. Furin cleavage site, FCS; porcine teschovirus-1 2A site, PTV-2A; signal peptide, SP; furin, F; signal peptide peptidase, SPP; endoplasmic reticulum, ER. (**B**) Schematic of *in vitro* testing of DNA constructs. (**C**) Cell-based ELISAs against 293Ts transfected with the indicated plasmid and stained with dilutions of an anti-influenza HA antibody, CR9114. (**D**) Confocal microscopy of 293T cells transfected with the indicated plasmid and stained with Hoescht 33342 (blue), anti-HA antibody, CR9114 (green), and anti-H5N1 NA polyclonal sera (red). (**E**) Cell-based ELISAs against 293Ts transfected with the indicated plasmid and stained with dilutions of an anti-N1 influenza polyclonal serum. (**F**) NA activity detected by ELLA using 293T cells transfected with the indicated plasmid. (**G**) Confocal microscopy of an HA fusion assay in 293T cells transfected with the indicated plasmids and stained with wheat germ agglutinin (WGA) to stain cell surfaces. For panels D and G, images are representative of two independent experiments and were taken of cells fixed 24 hours post-transfection. Scale bars = 30 µm. Panels C, E, and F are representative of four independent experiments, and data are shown as means ± SEM. Where indicated, significance was determined using a Kruskal-Wallis test followed by a Mann-Whitney *U* test with a Benjamini-Hochberg FDR correction. FDR-corrected *P*-values are reported above sample groups.

We have previously shown that expression of both NA and HA influenza virus glycoproteins from typical seasonal strains of influenza (H1N1, H3N2, IBV-Y, and IBV-V) in a single open reading frame can be an effective vaccine approach that elicits improved immune responses compared to split inactivated influenza vaccine platforms (22, 23). To understand if this approach could be useful as an H5N1 vaccine, we first generated a plasmid encoding for both the A/Texas/37/2024 NA and HA proteins from the same open reading frame by separating the two coding sequences via furin and PTV-2A cleavage sites (Fig. 1A). Following transfection of this NA-F2A-HA plasmid or a mixture of plasmids encoding for either protein alone into cells (NA + HA), alongside a luciferase-expressing control plasmid (Fig. 1B), we found that both NA and HA were expressed from the NA-F2A-HA plasmid (Fig. 1C and E) and detected on the surface of cells (Fig. 1D) to similar levels as when expressed individually. We also showed that the NA and HA expressed from our dual glycoprotein plasmid were not only detectable but also functionally active in NA-mediated removal of sialic acid from target proteins (Fig. 1F) and HA-mediated fusion of cell membranes under low pH conditions (Fig. 1G). These results suggest that this genetic approach can be used to deliver multiple H5N1 viral antigens to cells efficiently and effectively.

**Fig 2:**
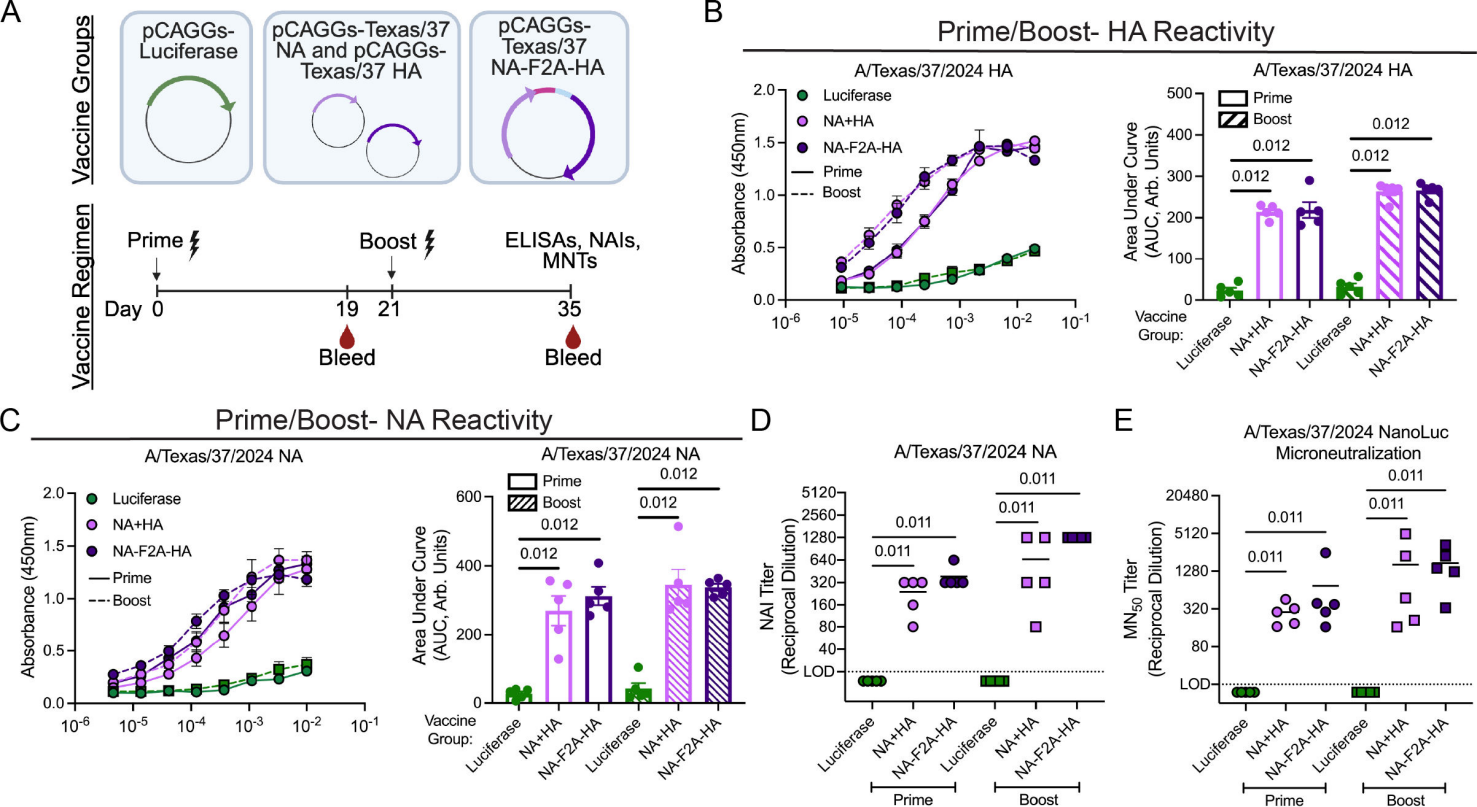
DNA vaccination of mice elicits sera reactivity and functionally inhibitory antibody responses. (**A**) Schematic detailing vaccine groups and regimens. (**B**) Cell-based ELISAs against 293Ts transfected with an A/Texas/37/2024 HA-expressing plasmid and stained with dilutions of sera from indicated groups of vaccinated mice following prime and boost (left) and area under the curve (AUC) analysis (right). (**C**) Cell-based ELISAs against 293Ts transfected with an A/Texas/37/2024 NA-expressing plasmid and stained with dilutions of sera from indicated groups of vaccinated mice, following prime and boost (left) and AUC analysis (right). (**D**) NAI assays using ELLAs performed with cells transfected with an A/Texas/37/2024 NA expression plasmid and sera from indicated groups of vaccinated mice following prime and boost. Values below the LOD (1:20 dilution, dotted line) were set to 15 for inclusion in the plot. (**E**) MN_50_ assays using an A/Texas/37/2024 reporter virus. Values below the LOD (1:20 dilution, dotted line) were set to 15 for inclusion in the plot. *n* = 5 mice per group shown, and mice were vaccinated with doses as follows: pCAGGs-Luciferase, 50 µg; pCAGGs NA, 23 µg; pCAGGs-HA, 27 µg; pCAGGs-NA-F2A-HA, 50 µg; for both prime and boost. All data are representative of two independent experiments. For panels B and C, data are shown as mean ± SEM. For panels D and E, lines indicate means. Statistical significance for panels B–D was determined using a Mann-Whitney *U* test with a Benjamini-Hochberg FDR correction. For panel E, a Kruskal-Wallis test followed by a Mann-Whitney *U* test with a Benjamini-Hochberg FDR correction was used for testing statistical significance. FDR-corrected *P*-values are reported above sample groups.

To investigate the immunogenicity of the A/Texas/37/2024 NA-F2A-HA vaccine, we electroporated either a luciferase control plasmid, a mixture of plasmids expressing either NA or HA, or our dual glycoprotein plasmid into animals (Fig. 2A). We rationalized that this approach would allow for rapid evaluation of the utility of the H5N1 NA-F2A-HA genomic configuration as a vaccine *in vivo*; in addition, DNA-vaccination approaches are currently used in several livestock vaccines (24–26), and therefore, if immunogenic, the DNA-based vaccines themselves could have utility in animal vaccination. Following both prime and boost, we found that our NA-F2A-HA vaccine elicited robust antibodies that recognized the A/Texas/37/2024 HA (Fig. 2B) or NA (Fig. 2C) antigens, in both cases to similar extents as a mixture of plasmids encoding for either protein. To test the functional neutralization capacity afforded by the delivery of NA in our plasmid, we performed neuraminidase inhibition (NAI) assays and found that antibodies generated by both our dual glycoprotein plasmid and a mixture of NA and HA-expressing plasmids were able to neutralize A/Texas/37/2024 NA activity *in vitro* (Fig. 2D). We also performed microneutralization (MN_50_) assays using an A/Texas/37/2024 reporter virus (A/Texas/37/2024 NanoLuc) to assess the function of HA-specific antibodies. We found that antibodies generated by both our dual glycoprotein plasmid and a mixture of NA and HA-expressing plasmids were able to efficiently neutralize multicycle A/Texas/37/2024 NanoLuc viral infection in MDCK cells (Fig. 2E).

**Fig 3:**
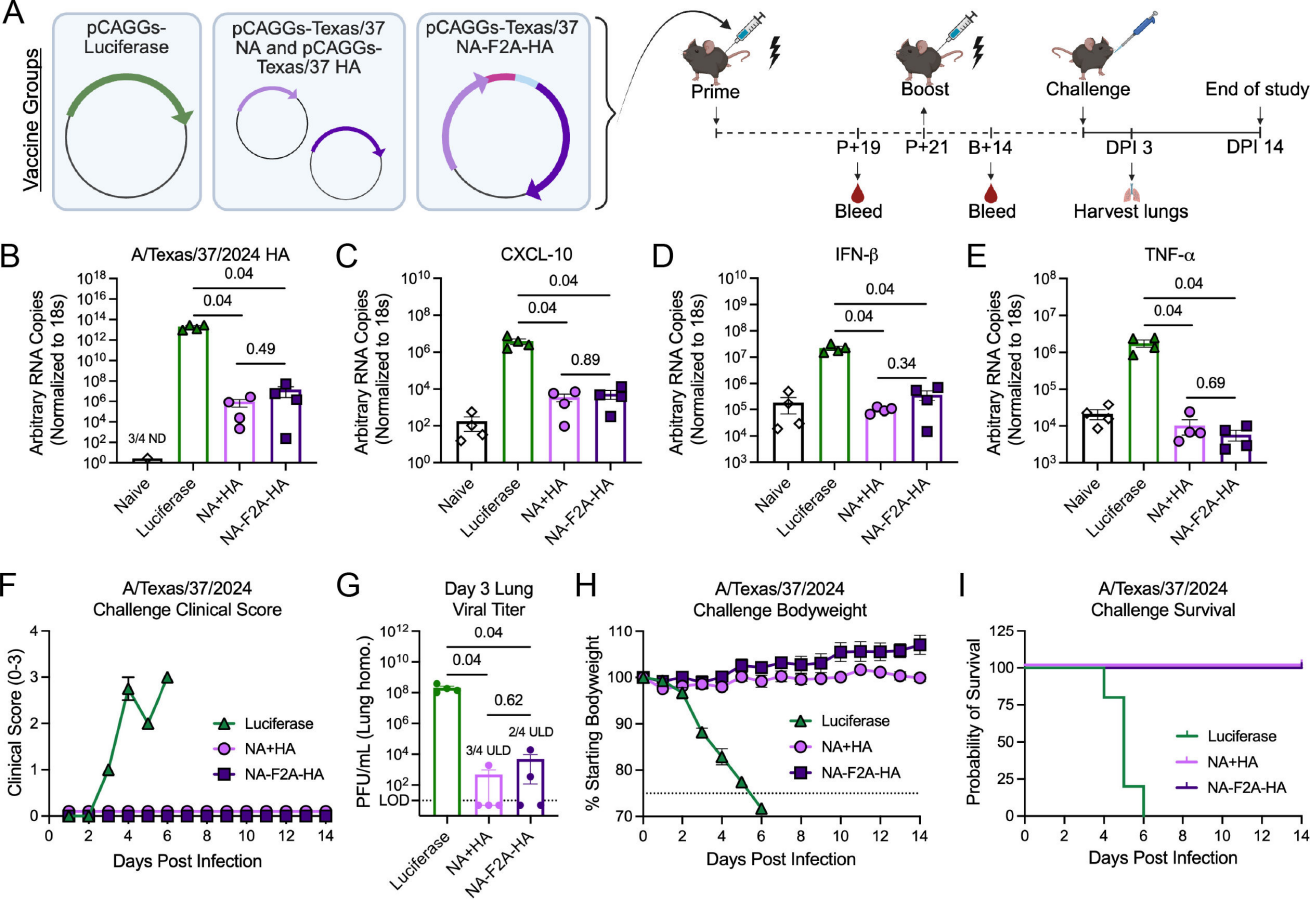
DNA vaccination protects from lethal challenge with A/Texas/37/2024. (**A**) Schematic detailing vaccine groups and vaccination/challenge regimen. Mice were primed with vaccine groups and bled to assess immune responses 19 days post-prime (P + 19). Mice were then boosted 21 days post-prime (P + 21) and bled 14 days later (B + 14). Groups were then challenged, and lungs were either taken 3 DPI or followed for body weight, survival, and clinical scores for 14 days. (**B–E**) qRT-PCR of lung homogenates taken from mice 3 DPI for (**B**) A/Texas/37/2024 HA, (**C**) CXCL-10, (**D**) IFN-β, and (**E**) TNF-⍺. (**F**) Clinical scores of vaccinated mice following lethal challenge with A/Texas/37/2024. (**G**) Viral lung titer of vaccinated mice 3 DPI determined using plaque assays on MDCK cells. The LOD (dotted line) is 10 PFU/mL, and values under the LOD (ULD) are shown as 5 PFU/mL for inclusion in the plot. (**H**) Change in body weight over 14 days post-viral challenge. Dotted line = 75% humane endpoint of loss of greater than 25% of starting body weight. (**I**) Percent survival of vaccinated mice following challenge with A/Texas/37/2024. *n* = 4 mice per group shown, and mice were vaccinated with doses as follows: pCAGGs-Luciferase, 50 µg; pCAGGs NA, 23 µg; pCAGGs-HA, 27 µg; pCAGGs-NA-F2A-HA 50 µg; for both prime and boost. All data are representative of two independent experiments and are shown as means ± SEM. Where indicated, statistical significance was determined using a Kruskal-Wallis test followed by a Mann-Whitney *U* test with a Benjamini-Hochberg FDR correction. FDR-corrected *P*-values are reported above sample groups.

To assess the protection from disease *in vivo* afforded by our NA-F2A-HA DNA plasmid, we challenged prime/boosted mice with WT A/Texas/37/2024 virus in a BSL-3 biocontainment facility and followed body weight, clinical scores, and viral load in tissues over the course of infection (Fig. 3A). We found significantly decreased levels of viral RNA in the lung homogenates of both the plasmid mixture and NA-F2A-HA-vaccinated mice compared to luciferase control-vaccinated animals at 3 days post-infection (DPI; Fig. 3B). Levels of cytokines and chemokines known to be associated with H5N1 infection in humans (C-X-C motif chemokine ligand 10 [CXCL-10], interferon beta [IFN-β], and tumor necrosis factor-alpha [TNF-⍺]) (27, 28) were also found to be significantly decreased in the NA-F2A-HA- and NA + HA-vaccinated mice compared to luciferase controls (Fig. 3C through E). This decreased inflammation was also reflected in clinical scores of mice over the course of infection, which revealed that our vaccine mitigated all signs of clinical disease compared to luciferase controls (Fig. 3F). Plaque assays using lung homogenates 3 days post-infection revealed that the viral replication in lungs was significantly decreased in both the NA-F2A-HA- and NA + HA-vaccinated groups, with multiple animals per group negative for infectious virus (Fig. 3G), suggesting that these vaccine-generated immune responses efficiently neutralized initial viral replication during early infection. Importantly, over the entire course of infection, NA-F2A-HA- and NA + HA-vaccinated mice were completely protected from severe morbidity and mortality with no noticeable weight loss, and NA-F2A-HA-vaccinated mice displayed a slight trend for faster weight gain (Fig. 3H and I).

**Fig 4:**
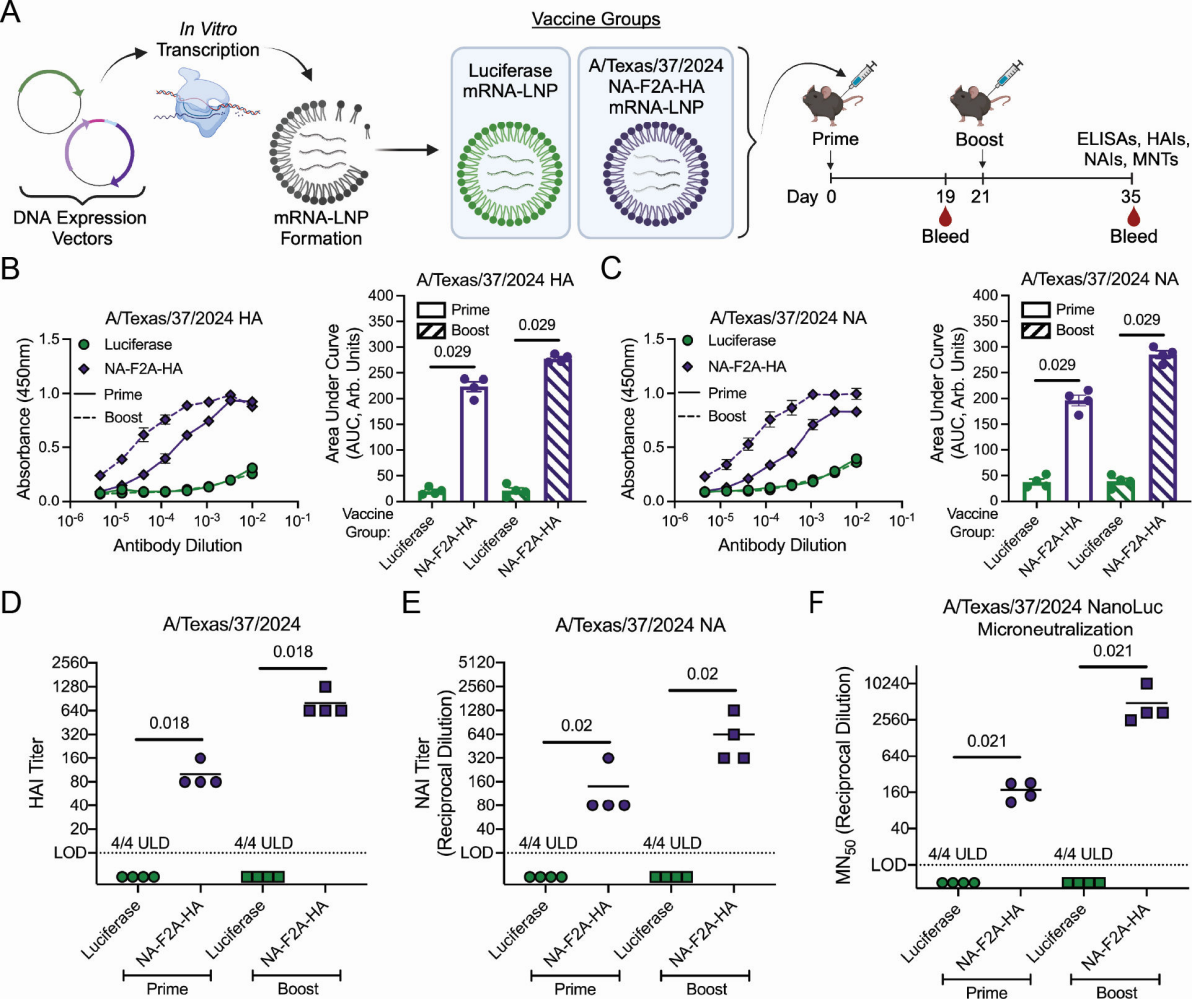
mRNA-LNP vaccination of mice elicits robust immune responses with functional neutralization activity. (**A**) Schematic detailing vaccine groups and regimens. (**B**) Cell-based ELISAs against 293Ts transfected with an A/Texas/37/2024 HA-expressing plasmid and stained with dilutions of sera from indicated groups of vaccinated mice following prime and boost (left) and AUC analysis (right). (**C**) Cell-based ELISAs against 293Ts transfected with an A/Texas/37/2024 NA-expressing plasmid and stained with dilutions of sera from indicated groups of vaccinated mice, following prime and boost (left) and AUC analysis (right). (**D**) HAI assay performed with A/Texas/37/2024 virus. Values below the limit of detection (LOD; 10 HAI units, dotted line) were set to five for inclusion in the plot. (**E**) NAIs using ELLAs performed with cells transfected with an A/Texas/37/2024 NA expression plasmid and sera from indicated groups of vaccinated mice following prime and boost. Values below the LOD (1:20 dilution, dotted line) were set to 10 for inclusion in the plot. (F) MN_50_ assays using an A/Texas/37/2024 reporter virus. Values below the LOD (1:20 dilution, dotted line) were set to 10 for inclusion in the plot. *n* = 4 mice per group shown, and mice were vaccinated with doses as follows: Luciferase mRNA-LNP 5 µg; NA-F2A-HA mRNA-LNP 5 µg; for both prime and boost. All data are representative of two independent experiments. For panels B and C, data are shown as mean ± SEM. For panels D–F, lines indicate means. Where indicated, statistical significance was determined using a Mann-Whitney *U* test with a Benjamini-Hochberg FDR correction. FDR-corrected *P*-values are reported above sample groups.

We next wanted to adapt this genetic configuration to an mRNA-LNP-based vaccine, as this nucleic acid vaccine formulation has been successfully used in humans and is the most likely path forward for H5N1 vaccines. We also developed our mRNA-LNP vaccine using generic LNPs using commercially available kits to ensure that results would not be dependent on any specific proprietary LNP formulations. NA-F2A-HA mRNA-LNPs were designed in the same way as with the DNA vectors (Fig. 4A) and delivered to mice in a prime/boost regimen to assess immunogenicity. The NA-F2A-HA mRNA-LNP was found to induce robust antibody responses to both NA and HA following both prime and boost (Fig. 4B and C), and these antibodies were found to have strong neutralizing activity against both proteins individually in hemagglutination inhibition (HAI) (Fig. 4D) and NAI assays (Fig. 4E). To assess their capacity for overall viral neutralization of A/Texas/37/2024, we performed cell-based microneutralization assays and found that the elicited antibodies were able to efficiently neutralize viral infection following both prime and boost (Fig. 4F).

**Fig 5:**
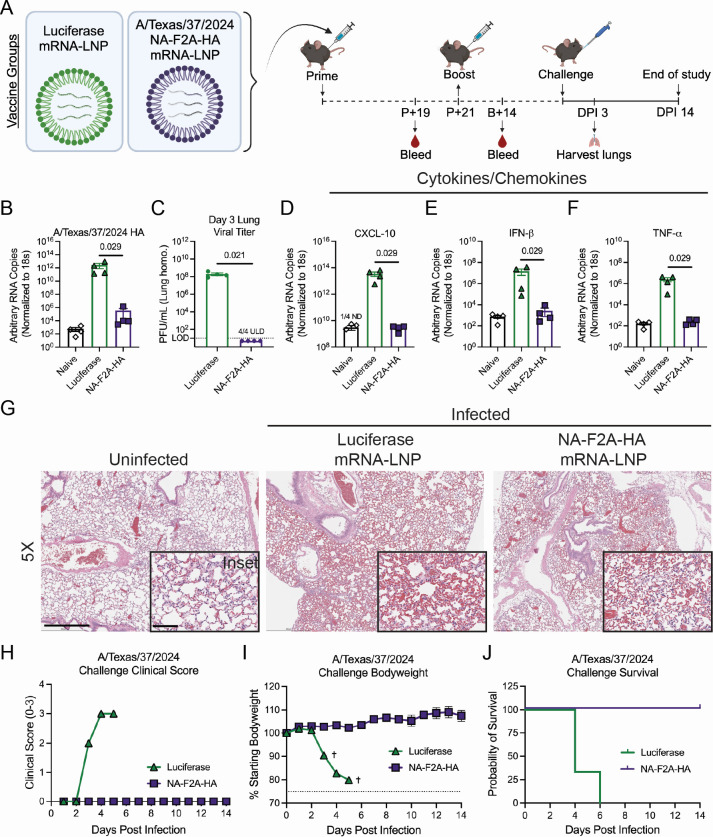
mRNA-LNP vaccination protects from lethal challenge with A/Texas/37/2024. (**A**) Schematic detailing vaccine groups and vaccination/challenge regimen. Mice were primed with vaccine groups and bled to assess immune responses 19 days post-prime (P + 19). Mice were then boosted 21 days post-prime (P + 21) and bled 14 days later (B + 14). Groups were then challenged, and lungs were either taken 3 DPI or followed for body weight, survival, and clinical scores for 14 days. (**B**) qRT-PCR of lung homogenates taken from mice 3 DPI for A/Texas/37/2024 HA. (**C**) Viral lung titer of vaccinated mice 3 DPI determined using plaque assays on MDCK cells. The LOD (dotted line) is 10 PFU/mL, and values under the LOD (ULD) are shown as 5 PFU/mL for inclusion in the plot. (**D–F**) qRT-PCR of lung homogenates taken from mice 3 DPI for (**D**) CXCL-10, (**E**) IFN-β, and (**F**) TNF-⍺. (**G**) H&E staining of lung sections of challenged mice taken at 3 DPI. Scale bar for 5× and inset = 500 µm and 100 µm, respectively. (**H**) Clinical scores of vaccinated mice following lethal challenge with A/Texas/37/2024. (**I**) Change in body weight over 14 days post-viral challenge. Dotted line = 75% humane endpoint of loss of greater than 25% of starting body weight. Crosses represent days in which one or more mice succumbed to infection as described in panel J. (**J**) Percent survival of vaccinated mice following challenge with A/Texas/37/2024. *n* = 4 mice per group shown, except control-vaccinated mice for panels **H–J** had *n* = 3, and mice were vaccinated with doses as follows: Luciferase mRNA-LNP 5 µg; NA-F2A-HA mRNA-LNP 5 µg; for both prime and boost. All data are representative of two independent experiments and are shown as means ± SEM. Where indicated, statistical significance was determined using a Mann-Whitney *U* test. *P*-values are reported above sample groups.

To understand the protection from disease conferred by the NA-F2A-HA mRNA-LNP vaccine, we then challenged boosted mice with A/Texas/37/2024 in a BSL-3 containment facility and again followed multiple measures of disease progression (Fig. 5A). We found that the amount of viral RNA was significantly decreased in the lungs of NA-F2A-HA mRNA-LNP-vaccinated mice compared to luciferase mRNA-LNP control vaccinated mice (Fig. 5B). Furthermore, in terms of infectious viral load, plaque assays revealed that while luciferase mRNA-LNP control vaccinated mice had high titers of virus in their lungs, NA-F2A-HA mRNA-LNP vaccinated mice all were below the limit of detection (LOD), suggesting the induction of neutralizing immunity (Fig. 5C). Additionally, the cytokines and chemokines CXCL-10, IFN-β, and TNF-⍺ were found to be drastically reduced in NA-F2A-HA mRNA-LNP vaccinated mouse lungs, suggesting little lung pathology and damage (Fig. 5D through F). Lung pathology of infected mice was examined using hematoxylin and eosin (H&E) staining, which showed limited immune cell infiltrate in both the control and NA-F2A-HA mRNA-LNP-vaccinated animals (Fig. 5G). These trends in pathology indicate the cause of death early in infection in control-vaccinated animals may be related to diffuse organ damage, which has been reported by multiple groups (29–32). Despite these similarities in pathology, luciferase mRNA-LNP control vaccinated animals experienced high clinical scores early during infection, while all signs of clinical disease were mitigated in NA-F2A-HA mRNA-LNP-vaccinated animals, further supporting the possibility of damage outside of the lungs (Fig. 5H). Ultimately, NA-F2A-HA mRNA-LNP vaccinated mice were found to be completely protected from body weight loss or morbidity during infection, while luciferase mRNA-LNP-vaccinated mice succumbed to infection by day 5 (Fig. 5I and J).

## DISCUSSION

In this study, we report the development of effective DNA and mRNA-LNP-based vaccines that encode the HA and NA glycoproteins of A/Texas/37/2024 (H5N1) and induce robust immune responses and protection against lethal viral challenge in a murine model. Our data show that both versions of the vaccines elicit high levels of HA- and NA-specific antibodies, functionally neutralizing activities, and effective viral clearance in the lungs, thereby preventing severe disease. This genetic configuration for the expression of both major viral glycoproteins and the demonstrated adaptability to different nucleic acid platforms suggests that this approach may be broadly useful for rapid vaccine development against emerging zoonotic influenza viruses with the potential to infect humans.

While our DNA-based experiments provided a rapid proof-of-concept approach for the unique genetic configuration of our vaccine, DNA-based vaccines themselves may represent a path forward for animal vaccination to help curb the circulation in both cattle and poultry. DNA vaccines are cost-effective, stable at ambient temperatures, and can be rapidly produced at scale, making them well-suited for deployment in agricultural settings (33–36). Moreover, they have been found to induce robust humoral and cellular immune responses in multiple different animals—including chickens against H5N1 avian influenza viruses and large animals such as horses—and they lack the risk of reversion to virulence, making them a safe alternative to traditional live-attenuated or inactivated vaccines (24, 26, 37, 38). By effectively preventing viral transmission among livestock, an effective DNA vaccine platform such as the one described here could limit zoonotic spillover and reduce the risk of human infection, ultimately safeguarding both the agricultural industry and public health.

The mRNA-LNP platform is highly likely to be utilized for human H5N1 vaccines as they have already been proven to be highly effective during the COVID-19 pandemic response; they produce both humoral and cellular immune responses, resulting in protection rates of over 90% (14, 39, 40). This efficacy has prompted the development of mRNA-LNP vaccines against both seasonal and emerging influenza viruses. In fact, several clinical trials have already been performed with seasonal influenza virus antigens demonstrating safety in humans as well as manufacturers’ commitment to the platform (41–43). The development of mRNA-LNP vaccines against pandemic influenza strains has also been of interest, with multiple groups reporting positive preclinical and clinical trial data of effective vaccines against H5 and H7 influenza viruses (44–51). While most of these studies have focused on HA-directed immunity only, those that have elicited NA-directed responses have seen improved protection against vaccine and drifted strains, strengthening the case for the inclusion of NA in vaccine designs to maximize and broaden protection (45, 46). The unique antigenic design of our vaccine represents an efficient and cost-effective way to deliver both NA and HA in future vaccine formulations designed to protect against H5N1 or other emerging viruses, streamlining the production of LNPs and affording maximal protection from disease. Furthermore, the platforms presented here represent highly adaptable strategies to quickly respond to novel emergent viral strains with the capacity to infect humans.

While the apparent efficacy of the vaccines described in this work is encouraging, our study has several limitations that should be noted. First, while mice provide a useful initial model for evaluating vaccine efficacy, they do not fully recapitulate immune responses observed in humans (52). For this reason, our findings should be validated in more clinically relevant animal models, such as ferrets, which more closely mimic the human course of influenza disease and have the ability to transmit the virus via respiratory droplets (53, 54). Additionally, our experiments were conducted in specific-pathogen-free mice, which do not account for the potential effects of pre-existing immunity from prior influenza exposure or vaccination, as well as the effects of previous or chronic non-influenza infections—immune statuses that are known to influence vaccine immunogenicity in real-world settings (55–58). Another important consideration to be explored is the long-term durability of responses induced by the mRNA vaccines. While influenza mRNA vaccines have been found to induce noninferior, and in some cases, superior, immune responses through 6 months in humans compared to inactivated vaccines, their longer-term durability remains incompletely understood (41). For SARS-CoV-2, mRNA vaccine-induced responses have been shown to wane substantially 6 months post-vaccination (59, 60). For this reason, it will be important in the future to perform long-term studies elucidating the durability of mRNA vaccine-induced responses to ensure protective antibody levels through an entire influenza season. Finally, while our vaccine provides robust protection against A/Texas/37/2024, it remains to be determined whether it confers cross-protection against antigenically distinct H5N1 strains that may pose future threats to both livestock and human populations.

Looking ahead, several key steps are necessary to translate these vaccines into viable countermeasures against emerging zoonotic influenza threats. A major advantage of our mRNA-LNP platform is its versatility and broad applicability. By using a generic LNP formulation, our platform has the potential to be rapidly adapted to combat emerging viral strains worldwide, regardless of regional manufacturing capabilities. Furthermore, our antigen configuration is compatible and effective with multiple LNP formulations, as demonstrated by the success of our previously published vaccine that utilized a different LNP platform. This adaptability broadens production possibilities to a variety of manufacturers and countries, facilitating more equitable and scalable vaccine distribution during global health crises. Additionally, the lipids used in our formulation are GMP-compatible and readily scalable, ensuring that large-scale production can be quickly achieved during outbreak response scenarios. Our DNA-based vaccine similarly represents a scalable strategy for preventing viral spread within cattle and poultry, mitigating zoonotic spillover risks. By integrating these strategies, both vaccine platforms presented here offer scalable and globally accessible solutions for mitigating the threat of the next influenza pandemic and equipping ourselves with effective tools to combat it.

## MATERIALS AND METHODS

### Cells

293T (CRL-3216) cells and MDCK (CCL-34) cells were both obtained from American Type Culture Collection and cultured at 37°C with 5% CO_2_. 293T cells were maintained with Dulbecco’s Modified Eagle Medium (DMEM, Gibco) that was supplemented with 5% fetal bovine serum (FBS) and GlutaMAX (Gibco), and MDCK cells were grown in Minimum Essential Medium (MEM, Gibco) with 5% FBS, GlutaMAX, sodium bicarbonate (Gibco), and HEPES (Gibco). All media used for cells was also supplemented with penicillin/streptomycin and Plasmocin prophylactic (Invitrogen, ANT-MPP).

### Viral strains

Wild-type A/Texas/37/2024 and reporter A/Texas/37/2024 NanoLuciferase viruses were rescued as previously described (61) under BSL-3 biocontainment conditions. The virus was then propagated on 10-day-old embryonated chicken eggs for 24 hours, then harvested, aliquoted, and frozen at −80°C. The virus was then titered on MDCK cells using plaque assays. All live virus work was approved by the institutional biosafety committee at Duke University. All segments from both viruses were amplified via SuperScript III One-Step RT-PCR systems (Thermo, 12574026), and cDNA was purified via gel purification. Viral cDNA was then Sanger sequenced to validate viral identity.

### Mouse vaccination

C57BL/6 mice (The Jackson Laboratory, #000664) were used in the study. DNA vaccination experiments, on day 0, the animals were anesthetized with ketamine and xylazine; and fur on the right hind leg was removed by shaving and treatment with hair removal cream. For initial vaccination, a solution of plasmid DNA (1 µg/µL, 50 µL) or an empty vector control plasmid solution (50 µL) was injected into the gastrocnemius muscle of the prepared leg using a syringe pump (PHD 2000, Harvard Apparatus), followed by percutaneous application of 8 electric pulses (70 V, 100 ms, 1 Hz) to the same muscle. The pulses were generated by the Square Wave Electroporation System (BTX ECM 830, Harvard Apparatus) and applied via a pair of platinum Tweezertrodes electrodes (BTX) that were pre-coated with an electrically conductive gel and separated by a 4 mm distance. On day 21, the same procedures above were repeated on the left hind leg for the boost dose. For both prime and boost, vaccine doses were as follows to deliver similar copies of DNA plasmids per group: pCAGGs-Luciferase, 50 µg; pCAGGs-Texas/24 NA, 23 µg; and pCAGGs-Texas/24 HA, 27 µg; pCAGGs-Texas/24 NA-F2A-HA, 50 µg. The serum from animals was collected on days 19 (post-prime sera) and 35 (post-boost sera) for measurement of immune responses. For mRNA-LNP vaccination, mice were vaccinated intramuscularly with 5 µg of each indicated mRNA-LNP, diluted in pharmaceutical-grade phosphate-buffered saline (PBS). Mice were primed, boosted, and bled for sera in the same design as for DNA vaccinations.

### Mouse viral challenge

For viral challenge, between ~2.5 and 8 weeks post-boost, mice were anesthetized with ketamine/xylazine and intranasally inoculated with 200 PFU of WT A/Texas/37/2024 diluted in pharmaceutical-grade PBS. Body weight and clinical score were monitored for 14 days post-infection, or for groups in which lungs were obtained, lungs were harvested according to protocol at 3 days post-infection, homogenized in 1 mL pharmaceutical-grade PBS, and aliquoted. Clinical scores were defined as the following: 0 = normal activity, no signs of illness; 1 = mild lethargy and hunched body posture; 2 = moderate lethargy, hunched body posture, ears pointed back, and mouse still mobile if provoked or unprovoked; 3 = severe lethargy, hunched posture with ears pointed back, and eyes squinted, little to no movement (even if provoked). If mice fell below the predetermined humane endpoint of 25% body weight loss from pre-challenge weights, they were sacrificed according to Duke University Institutional Animal Care and Use Committee (IACUC)-approved protocols.

### Plasmids

The A/Texas/37/2024 HA and NA sequences were obtained from the following accession numbers through GISAID (https://gisaid.org/): NA, EPI3171486; HA, EPI3171488. In the case that single nucleotides were missing from entries, they were inferred from other H5N1 strains. Nucleotide sequences were human codon-optimized and ordered as complete genomic segments from Integrated DNA Technologies (IDT) that were then cloned into pCAGGS expression vectors as previously described (22), and sequence identity was confirmed using Sanger sequencing for DNA vaccine studies.

### *In vitro* mRNA transcription

The NA-F2A-HA DNA construct containing a T7 promoter, 5′ and 3′ UTR regions, and a 110-nucleotide poly (A) tail was codon optimized and synthesized at GenScript (Singapore) and subcloned into a pUC-based plasmid with kanamycin-resistant gene. The capped nucleoside-modified (N1-methylpseudouridine, m1Ψ) mRNA was synthesized using the co-transcriptional capping method with CleanCap AG trimer and m1Ψ (TriLink, USA) by Trilink protocol (Cat No. N-7113). The mRNA was purified using oligo-dT affinity monolith chromatography (Sartorius, Germany). Length and mRNA integrity were assessed using the native agarose gel. The mRNA was stored frozen (1 mg/mL) at −80°C in nuclease-free water.

### mRNA-LNP vaccine formulation

The mRNA-LNP vaccines were formulated by encapsulation using a GenVoy ionizable lipid mixture (GenVoy-ILM) and NanoAssemblr Ignite machine (Precision NanoSystems [PNI], CA, USA). All procedures follow the manufacturer’s instructions. GenVoy-ILM lipid mixture consists of PNI Ionizable Lipid: DSPC:Cholesterol:PNI stabilizer at 50:10:37.5:2.5 mol%. The formulation was prepared on the Ignite machine with a program set for mixing of GenVoy-ILM and mRNA at a molar ratio of 4:1 with a total flow rate of 12 mL/min and a flow ratio of 3:1. The LNP encapsulated mRNA was buffer exchanged to PBS using 30 kDa, Amicon ultra centrifugal filters (Merck, Germany). Particle size was measured by Dynamic Light Scattering using Zetasizer Ultra machine (Malvern Panalytical, UK). Encapsulation efficiency (EE) and mRNA concentration were determined using Quant-iT RiboGreen RNA Assay (Thermo Fisher, USA). The EE was calculated according to the following formula: EE% = (total RNA − free RNA)/total RNA.

### Cell-based enzyme-linked immunosorbance assays

For cell-based enzyme-linked immunosorbance assays (ELISAs), 293T cells were first plated in poly-L-lysine-treated 96-well plates. Cells were then transfected with the indicated plasmid in Opti-MEM (Gibco) using Trans-IT-LT1 (Mirus Bio, #2304) and left to incubate at 37°C with 5% CO_2_. After 24 hours, cells were fixed using 2% paraformaldehyde (PFA) and washed twice with 1× PBS. Cells were blocked in 3% non-fat milk in 1× PBS for at least 2 hours, then dilutions of sera/antibody in the blocking solution were added and allowed to incubate overnight at 4°C. Cells were washed four times with 1× PBS, then a secondary antibody (goat anti-mouse horseradish peroxidase [HRP] [Invitrogen, #A16072] or goat anti-human HRP [Invitrogen, #A18805]) diluted 1:10,000 was added to each well. Plates were finally washed four times again with 1× PBS before 1-Step TMB ELISA Substrate Solutions (Fisher Scientific, #PI34028) were added to each well to allow the signal to develop. The reaction was then stopped with 1M H_2_SO_4_, and absorbance was read at 450 nm on a Varioskan LUX plate reader. Data were analyzed using Prism 9 (GraphPad), and area under the curve analyses were calculated based on dilutions up to the point of reaching a signal plateau.

### Confocal microscopy

For confocal microscopy, 293T cells were plated onto poly-L-lysine-treated coverslips in 24-well plates, then transfected with the indicated plasmid in Opti-MEM using Trans-IT-LT1 transfection reagent and left to incubate overnight at 37°C with 5% CO_2_. Coverslips were then gently washed once with 1× PBS and then fixed using 4% PFA. Coverslips were washed twice with 1× PBS and then blocked with 5% bovine serum albumin (BSA) in 1× PBS for at least 1 hour at room temperature. Primary antibody (anti-HA CR9114, generated by the Moody lab at Duke University School of Medicine, 10 µg/mL; and anti-A/Texas/37/2024 NA mouse polyclonal sera, 1:500) was then diluted in 0.5% BSA in PBS and added to coverslips, which were then left to incubate at 4°C overnight. Coverslips were washed three times with 1× PBS, then a secondary antibody (goat-anti human Alexa Fluor488 [Invitrogen, #A11013] or goat-anti mouse Alexa Fluor647 [Invitrogen, #A21235]) was diluted 1:10,000 and added to coverslips and allowed to incubate for 1 hour at room temperature. Coverslips were washed three times again with PBS, then stained for nuclei with Hoechst 3342 (Life Technologies, 1:2,500), and mounted onto slides (Prolong Diamond). Slides were imaged using an inverted IX83 Olympus confocal microscope with a motorized XY-stage (Prior), and images were processed identically using ImageJ software (NIH).

### HA fusion assay

For HA fusion assays, 293T cells were plated onto poly-L-lysine-treated coverslips in 24-well plates, then transfected with the indicated plasmid in Opti-MEM using TransIT-LT1 and allowed to incubate at 37°C with 5% CO_2_ for 24 hours. The next day, the cell medium was replaced with Opti-MEM supplemented with 0.35% BSA, 0.01% FBS, and 5% penicillin/streptomycin with 1 mg/mL L-1-tosylamido-2-phenylethyl chloromethyl ketone (TPCK)-treated trypsin for 20 minutes at 37°C with 5% CO_2_. Cells were then treated with either DMEM at physiological pH = 7.4 or DMEM acidified with citric acid at pH = 5 for 20 minutes at 37°C with 5% CO_2_. All medium was then replaced with fresh DMEM at physiological pH and allowed to recover at 37°C for 4 hours. Coverslips were then fixed with 4% PFA and stained with 20 µg/mL wheat germ agglutinin (WGA, Vector Laboratories) fluorescent lectin and Hoechst 33342 in PBS for 1 hour at room temperature. Coverslips were then mounted onto slides and imaged as described for confocal microscopy.

### Enzyme-linked lectin assays

For enzyme-linked lectin assays (ELLAs) to determine neuraminidase activity, wells of immunograde 96-well plates were coated with 25 µg/mL fetuin (Sigma, F3385) in 1× coating buffer (KPL) for at least 24 hours at 4°C. 293T cells were plated and transfected with the indicated plasmids in Opti-MEM using TransIT-LT1 and left to incubate at 37°C. After 24 hours, cells were resuspended in Opti-MEM supplemented with 0.35% BSA, 0.01% FBS, and 5% penicillin/streptomycin, counted, and normalized across samples. Fetuin-coated plates were washed three times with 1× PBS-T, and cell suspensions were added. Plates were firmly sealed and left to incubate at 37°C with 5% CO_2_ for 18 hours. Plates were then washed three times with 1× PBS-T before peanut agglutinin-HRP (1 µg/mL, Sigma) was diluted in sample PBS with 0.5% Tween-20 and added to plates for 2 hours in the dark at room temperature. Plates were washed three times again with 1× PBS-T, and the signal was developed as previously described for ELISAs.

### NAI assays

For NAI assays, a similar protocol to ELLAs was followed. To generate recombinant A/Texas/37/2024 neuraminidase, 293T cells were transfected with pCAGGs-A/Texas/37/2024 NA plasmid in Opti-MEM using Lipofectamine 3000, and at 24 hours post-transfection, cells were harvested using 1× PBS and counted. Cells were brought to a concentration of 1.2 × 10^5^ cells/mL, spun down, and resuspended in equal volume PBS with 0.5% Tween-20 to lyse the cells. Sera from mice was treated at a 1:4 dilution with receptor-destroying enzyme (RDE, Denka Seiken #370013) for 18 hours at 37°C, followed by inactivation of RDE enzyme with treatment at 56°C for 45 minutes. Sera was then serially diluted in PBS and added to fetuin-coated plates that had been washed three times with PBS/0.01% Tween-20 (PBS-T). Cell lysates were then thoroughly mixed and added equally to each well, and plates were left to incubate at 37°C for 18 hours. Wells including only cell lysate were included as positive controls. Plates were then developed using peanut agglutinin-HRPO secondary antibody and 1-Step TMB Substrate as described for ELLAs. NA activity was calculated as the following percentage: [(serum absorbance − mean background absorbance)/(mean positive control absorbance − mean background absorbance)] × 100. NAI titer was reported as the reciprocal of the lowest dilution of sera that inhibited greater than or equal to 50% of NA-only positive control activity.

### Microneutralization assays

All MN_50_ assays were performed in the BSL-3 biocontainment facility. RDE-treated sera were serially diluted in complete MEM media, and an equal volume of a dilution of A/Texas/37/2024 NanoLuc virus was added to each well except negative control wells. The virus-serum mixtures were left to incubate for 1 hour at 37°C with 5% CO_2_ and were then added to 96-well plates containing plated MDCK cells. Cells with sera/virus dilutions were then left to incubate for 24 hours at 37°C with 5% CO_2_. The next day, cells were washed once with 1× PBS and then were lysed in Luciferase Cell Culture Lysis Reagent (Promega, E1531) for 10 minutes at room temperature. Cell lysates were then added to a white 96-well plate, and the signal was developed using the Nano-Glo Luciferase Assay System (Promega, N1130), and bioluminescence was quantified using a BioTek Synergy LX plate reader. To calculate the MN_50_ titer, nonlinear regressions based on sera dilutions were calculated for each sample, and the dilution at which each sample reached a 50% value of the positive control infected well value was reported as the MN_50_ value (Prism 9, GraphPad).

### HAI assays

For HAI assays, RDE-treated sera was diluted 1:10 in PBS, then serially diluted 1:2 in 96-well V-bottom microplates. Equal amounts of standardized dilutions of A/Texas/37/2024 virus (4 HA units) were then added to each sera dilution and allowed to incubate at room temperature for 15 minutes. A standardized solution of 1% horse red blood cells (RBCs; Lampire Biological Laboratories, #7203401) in PBS was then added on top of the virus/sera mixtures, and the plates were covered and allowed to settle at 4°C overnight. The next day, HAI titers were calculated as the reciprocal of the last dilution of sera that inhibited hemagglutination, which was indicated by a clear RBC pellet.

### Plaque assays

All plaque assays were performed in the BSL-3 biocontainment facility. For plaque assays, MDCK cells were plated in complete MEM in six-well plates. After 24 hours, cells were washed once with 1× PBS, and lung homogenates from infected mice were diluted in PBS/5% BSA and then added to plates for 1 hour at 37°C with 5% CO_2_. After 1 hour, virus dilutions were aspirated and replaced with an agar overlay (MEM, GlutaMAX, sodium bicarbonate, HEPES, penicillin/streptomycin, BSA, DEAE-dextran, 1.2% cellulose colloidal microcrystalline [Sigma, 43244] with 1 µg/mL TPCK-treated trypsin). After 48 hours post-infection, overlays were aspirated, and wells were stained with 0.1% crystal violet in 10% neutral buffered formalin (NBF) before plaques were counted to determine titer.

### RNA extraction and qRT-PCR

Viral RNA was extracted using TRIzol Reagent (Invitrogen, 15596026) in Phasemaker tubes (Thermo, A33248). Quantitative reverse transcription-PCR (qRT-PCR) was then performed using the following probes: a custom primer/probe set ordered from IDT targeting the A/Texas/37/2024 HA RNA (forward: TTACACATGCCCAAGACATACT, reverse: CAGCTACACTGCAGTCCTTTA, and probe: /56-FAM/AACACACAA/ZEN/CGGGAAGCTATGCGA/3IABkFQ); commercial TaqMan probes for CXCL-10 (Mm00445235_m1), IFN-β (Mm00439552_s1), and TNF-⍺ (Mm00443258_m1) and normalized to endogenous 18S RNA (Applied Biosystems 4318839). qRT-PCR was performed using the EXPRESS One-Step Superscript qRT-PCR kit (Invitrogen, 11781200). Samples were analyzed on an Applied Biosystems QuantStudio 3 Real-Time PCR System.

### Lung histology

Mouse lungs were obtained from mice at 3 days post-infection in accordance with Duke IACUC protocols and were fixed in 10% NBF for at least 48 hours. Samples were then sent to HistoWiz Laboratories where they were paraffin embedded and sectioned onto slides before being stained with hematoxylin and eosin for imaging.

### Graphing and statistics

Experimental data were graphed using Prism 9 (GraphPad Software), and statistical analysis was performed using R statistical software (R Foundation for Statistical Computing, Vienna, Austria). Comparisons between two groups were determined using a Mann-Whitney *U* test, and if there was more than one comparison being made, a Benjamini-Hochberg false discovery rate (FDR) correction was applied. Comparisons between more than two groups were first performed using a Kruskal-Wallis test, and if the resulting *P* value was <0.05, a Mann-Whitney *U* test was performed to compare pairs of groups. The Benjamini-Hochberg procedure was then applied within each figure panel to control for the FDR for multiple comparisons. All tests were two-sided, with an alpha level of 0.05. All experiments were performed at least twice as separate, independent replicates. Values not detected (ND) in an assay are indicated as ND and were assigned arbitrary values below the limit of detection for inclusion in graph and statistical analysis. Schematics in figures were created using BioRender.

## Data Availability

The data included in this study are displayed in the figures, and no large data sets (sequencing, etc.) which would typically be deposited into public repositories were generated. Any requests for the data underlying the graphs shown will be fulfilled by the corresponding author.
